# Machine learning provides specific detection of salt and drought stresses in cucumber based on miRNA characteristics

**DOI:** 10.1186/s13007-023-01095-x

**Published:** 2023-11-08

**Authors:** Parvin Mohammadi, Keyvan Asefpour Vakilian

**Affiliations:** 1 Department of Agrotechnology, College of Abouraihan, University of Tehran, Tehran, Iran; 2 Department of Biosystems Engineering, Gorgan University of Agricultural Sciences and Natural Resources, Gorgan, Iran

**Keywords:** Support vector machine, Optimization algorithms, miRNA biosensor, Image textural features

## Abstract

**Background:**

Specific detection of the type and severity of plant abiotic stresses helps prevent yield loss by considering timely actions. This study introduces a novel method to detect the type and severity of stress in cucumber plants under salinity and drought conditions. Various features, i.e., morphological (image textural features), physiological/biochemical (relative water content, chlorophyll, catalase activity, anthocyanins, phenol content, and proline), as well as miRNA characteristics (the concentration of miRNA-156a, miRNA-166i, miRNA-399g, and miRNA-477b) were extracted from plant leaves, and machine learning methods were used to predict the type and severity of stress by having these features. Support vector machine (SVM) with parameters optimized by genetic algorithm (GA) and particle swarm optimization (PSO) was used for machine learning.

**Results:**

The coefficient of determination of predicting the stress type and severity in plants under both stresses was 0.61, 0.82, and 0.99 using morphological, physiological/biochemical, and miRNA characteristics, respectively. This reveals machine learning methods optimized by metaheuristic optimization techniques can provide specific detection of salt and drought stresses in cucumber plants based on miRNA characteristics. Among the study miRNAs, miRNA-477b and miRNA-399g had the highest and lowest contribution to salt and drought stresses, respectively.

**Conclusions:**

Comapred to conventional plant traits, miRNAs are more reliable features for providing us with valuable information about plant abiotic diseases at early stages. Using an electrochemical miRNA biosensor similar to one used in this work to measure the miRNA concentration in plant leaves and using a machine learning algorithm such as SVM enable farmers to detect the salt and drought stress at early stages in cucumber plants with very high accuracy.

**Supplementary Information:**

The online version contains supplementary material available at 10.1186/s13007-023-01095-x.

## Background

Although the morphological, physiological, and biochemical characteristics of plants are significantly affected by biotic and abiotic stresses, their variations are not specific to the stress [2]. For example, different sources of stress can result in similar changes in plant height and root and shoot weights as plant morphological features. For a comprehensive understanding of plant response at the molecular and cellular level in stress-induced studies, investigating the traits that vary almost specific to the stress is favorable. Today, early detection of plant biotic and abiotic stresses has been studied by examining the expression of miRNAs in many crop plants [57].

As small non-coding RNA molecules in living organisms, miRNAs are 17 to 23 nucleotides in length and have significant effects on gene expression [25]. The targets of plant miRNAs are mainly signal enzymes and proteins involved in physiological and biochemical processes such as plant growth, regulation of plant hormones, and signal transduction [43, 75]. miRNAs not only involve plant abiotic and biotic stress responses [44], they have crucial roles in all fields of plant physiology, such as plant hormonal regulation [16] and developmental processes [8]. Light, temperature, nutrient deficiency and toxicity, drought, salinity, and carbon dioxide are common sources of plant abiotic stresses [23].

Plant stress-involved miRNA expression is mainly both spatial- and temporal-specific [42]. Identifying plant stress-responsive miRNAs helps increase our knowledge about their role in the improvement of the plant stress tolerance mechanism [86]. In fact, to understand stress tolerance in plants, it is essential to investigate miRNA-mediated gene regulatory networks that control biological processes, such as responses to the environment [22]. The roles of miRNA-target gene structures in regulating plant stress responses have been extensively reported during the last decade [21]. Studies indicate that conserved miRNA families such as miR156, miR159, miR160, miR167, miR172, miR319, miR393, miR395, miR398, and miR399 respond remarkably toward abiotic stress [86]. Recent studies have reported that some of these miRNAs exert an up(down-regulation behavior toward several plant stresses, while some miRNAs are influenced by only one stress or at least only one of their roles has been characterized [2, 60].

Salt and drought stresses are among the undesirable abiotic stresses that can influence crop production, and their severe conditions affect the physiological and biochemical properties of a large number of plants significantly [13, 50]. Plant response toward drought is studied considering physiological properties, e.g., relative water content and antioxidative enzymatic responses [11]. Salt stress influences plant growth and development through osmotic stress and ion toxicity [12]. Crops adapt to salinity by inducing changes during transcription and translational levels [81].

Cucumber (*Cucumis sativus* L.) is strongly sensitive to drought and salinity, particularly at the early stages of growth [85]. Severe salinity and drought significantly affect plant growth, photosynthesis, biochemistry, and texture of fruits in cucumbers [58]. Various types of protein synthesis and gene transcription occur during the plant growth and development of cucumbers, which are affected by abiotic stresses. For instance, the biosynthesis and the drought resistance change greatly by the unconventional expression of *CsCER1* in cucumber plants [77]. *CsDCLs*, *CsAGOs*, and *CsRDRs* generally respond to abiotic stresses in cucumbers [20]. Investigating the effects of drought and salt stress on molecular regulation during seed germination and seedling growth has resulted in providing deep sequencing data of miRNA expression in cucumber plants. Du et al. [18] used proteomics and transcriptomics analysis to investigate the plant response toward salinity and drought during the post-germinative development in cucumber plants. They reported that the study stresses caused differential expression of 36 miRNAs and 768 proteins compared with the control, of which four miRNAs had similar patterns by both stresses: miR156a, miR166i, miR399g, and miR477b. Therefore, monitoring these miRNAs during exposure to drought and salinity stresses is capable of providing us with useful information about the physiology of cucumbers.

Several techniques, including microarrays [82], northern blotting [74], and polymerase chain reaction (PCR) [48], have been extensively used to investigate the influence of stress on plant miRNA functions. Nonetheless, these techniques suffer from various limitations, e.g., an unfavorable detection limit, a small linear range, and low sensitivity [52, 73]. Instead, sensors that have a living biological receptor and are called biosensors have been developed as a reliable analytical technique for the accurate and sensitive detection of miRNA concentration [33]. Constructing low-cost and portable electrochemical biosensors for the specific and sensitive detection of miRNAs that are involved in the salt and drought stresses of cucumber seems to be useful for studying the response of the plant at the molecular level. Today, hundreds of electrochemical biosensors with acceptable specificity and wide linear range have been introduced to measure the concentration of miRNAs in attomolar and femtomolar levels [17, 71]).

Supervised and unsupervised machine learning methods have been developed extensively in the field of plant science to model plant behavior during planting, cultivation, maintenance, harvesting, and post-harvest [19, 28, 32]. The machine is capable of learning the complex multivariate relationships between inputs and output using the training data [64]. The application of machine learning for computational analysis of the role of miRNAs toward plant biotic and abiotic stresses is growing since we are faced with big data and large datasets in these problems. Recently, Meher et al. [51] and Pradhan et al. [62] offered software applications for predicting various abiotic stress-specific miRNAs using features derived from miRNA sequences. They reported that the introduced tools could be efficiently utilized for large-scale prediction of abiotic stress-specific miRNAs using only sequence information.

In case of obtaining a dataset, including morphological, physiological, biochemical, as well as miRNA concentrations of cucumber plants under salt and drought stresses, there is an important question: which of these features should be trained to machine learning techniques for the reliable detection of type and severity of the stress? Therefore, in this study, salt and drought stresses at different levels are applied to young cucumber plants individually and simultaneously, and various traits of the plants were measured during the experiments to create a dataset useful for training the machine. Then, support vector machine (SVM), optimized by metaheuristic optimization algorithms, i.e., genetic algorithm (GA) and particle swarm optimization (PSO), was utilized for the prediction of the stress type and severity using the dataset. Hence, the objectives of this study are: (a) to find the performance of each morphological, physiological, and biochemical trait, as well as miRNA concentrations for the detection of salt and drought stresses in cucumber plants, and (b) to determine a reliable machine learning algorithm optimized by sophisticated evolutionary and swarm-based methods for the specific detection of stress.

## Material and methods

**Fig. 1:**
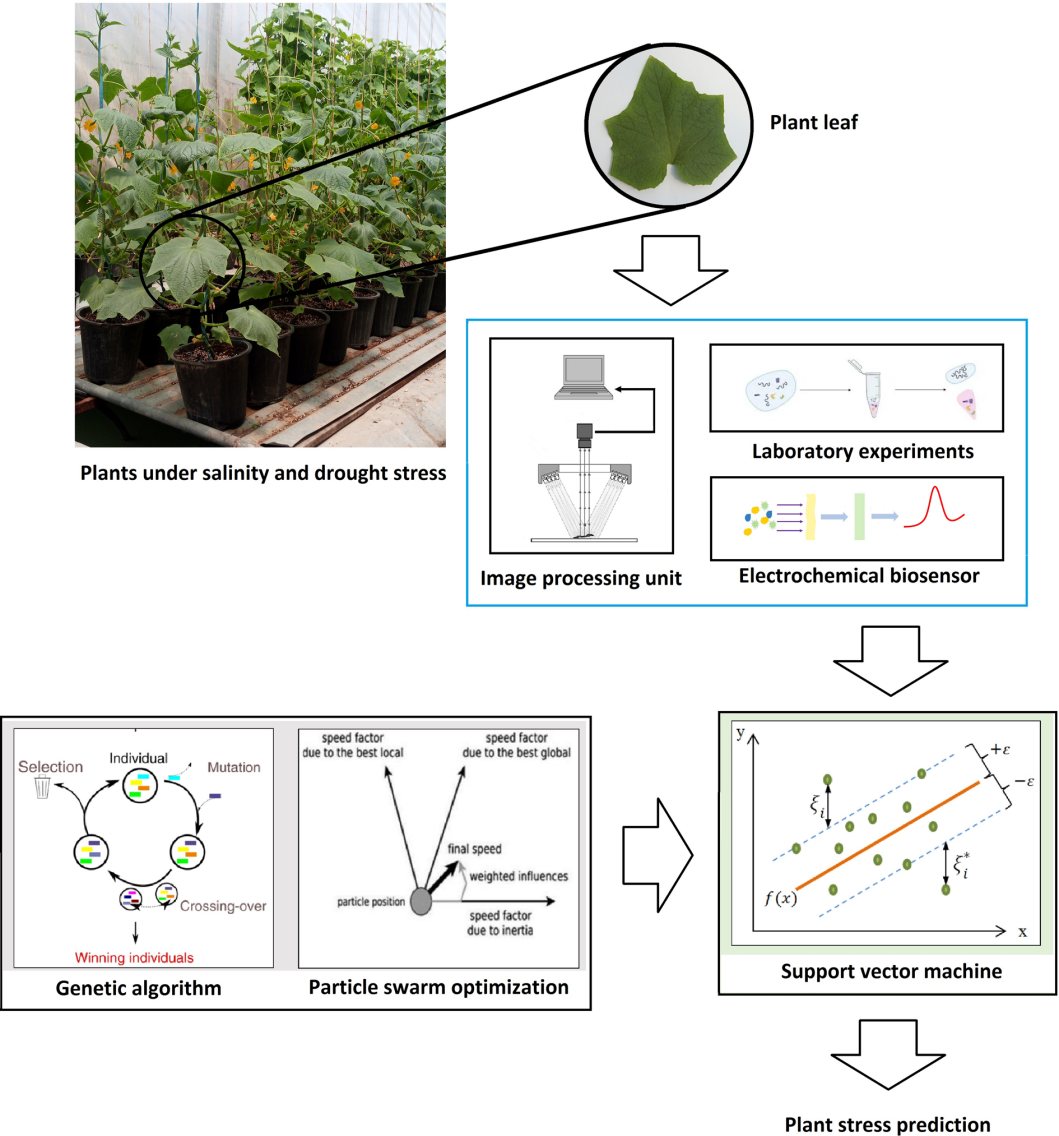
Flowchart of the present study

Figure 1 shows the flowchart of the present study. Morphological features, including energy, entropy, and homogeneity of images captured from plant leaves, were extracted using an image processing unit. Laboratory experiments were conducted to measure relative water content, chlorophyll, catalase activity, anthocyanins, phenol content, and proline in plant leaves. An electrochemical miRNA biosensor, introduced by Hakimian and Ghourchian [26], was used to measure miRNAs in the plant samples. In this scheme, a thiolated oligonucleotide probe is immobilized on the surface of a gold working electrode. The probe is then located in the sample to conduct probe-target hybridization. As electroactive labels, positively-charged polyethyleneimine-silver nanoparticles are then absorbed onto the hybridization product, which is negatively charged. The anodic peak current obtained due to the oxidation of silver nanoparticles will be proportional to the target miRNA concentration. Then, extracted features (as machine inputs) were trained to SVM, the parameters of which were optimized by GA and PSO. The machine outputs were the type and severity of the stress.

### Plant material and experimental design

Cucumber seeds were purchased from a local store. After surface sterilizing with NaClO and rinsing with distilled water thoroughly, the seeds were located in plug trays containing vermiculite: peat: perlite (3: 2: 1). After 3 weeks, seedlings were transferred to 5 L pots with a similar growth medium of plug trays. The light intensity of the growth room was 120 μmolm^−2^ s^−1^ with a relative humidity of 70 ± 5%. Tap water was supplied daily for the pots to provide moisture content near the field capacity.

After two months, treatments were applied: although daily tap water supply was continued for the control plants (*W*_0_), drought-treated plants were faced with withholding water at 80% (*W*_1_), 60% (*W*_2_), and 40% (*W*_3_) of field capacity to investigate various levels of stress severity. Salinity-treated plants were irrigated with water containing various concentrations of NaCl, i.e., 0 (control, *S*_0_), 20 mM (*S*_1_), 40 mM (*S*_2_), and 60 mM (*S*_3_). Therefore, plants were treated with salt and drought simultaneously, each at four levels, and since the experiments were performed with three replications, 4 × 4 × 3 = 48 pots were used in this study. Plant features were measured five times at 3 day intervals beginning with applying the stress by transferring three leave, randomly selected from each pot, to the laboratory for further analysis.

### Morpho-physiological and biochemical measurements

#### Morphological characteristics

Digital image processing was used to extract the morphological features of the leaves. A CCD digital camera and a 200-LEDs lighting array to increase light uniformity were used for image acquisition. Leaves were extracted from the background in the captured images through Canny edge detection [6]. After converting the color images to grayscale images, the gray-level cooccurrence matrix (GLCM) was utilized to measure the spatial dependence of gray-level values [7, 31]. An arbitrary element (*i,j*) in GLCM demonstrates the number of times that the pixel with a gray-level value *i* is located adjacent to a pixel with a gray-level value *j*. Recent studies have revealed that three image textural features, i.e., entropy (Eq. 1), energy (Eq. 2), and local homogeneity (Eq. 3), are useful for identifying plant health status [68]

1 $${\text{Entropy}} = - \sum\limits_{i} {\sum\limits_{j} {p(i,j)\log (p(i,j))} }$$

2 $${\text{Energy}} = \sum\limits_{i} {\sum\limits_{j} {p(i,j)^{2} } }$$

3 $${\text{Local homogeneity}} = \sum\limits_{i} {\sum\limits_{j} {\frac{p(i,j)}{{1 + (i - j)^{2} }}} }$$

where *p*(*i*,*j*) is the (*i*,*j*)-th element of the GLCM. The code for image processing was written in the MATLAB R2018b programming environment.

#### Physiological and biochemical characteristics

The relative water content (RWC) of the plant leaves was calculated according to Goñi et al. [24] by having the fresh weight, turgid weight, and dry weight. The chlorophyll index (CI) in the leaves was measured using a spectral chlorophyll meter (CM1000 Spectrum Technologies, USA). Catalase (EC. 1.11.1.6) activity (CA), Anthocyanin content (AC), phenol content (PhC), and proline content (PC) in the leaf samples were measured according to Havir and McHale [29], Mancinelli [47], Singleton and Rossi [67], and Bates et al. [10], respectively.

#### miRNA concentrations

Total RNA was isolated from 0.05 g of the leaves, according to Yamaguchi et al. [79]. A three-electrode electrochemical biosensor was used to measure the stress-involved miRNA concentrations in the isolated total RNA samples. Table 1 shows a list of the target miRNAs investigated in this study and their sequence. The working, reference, and auxiliary electrodes used in the biosensor were Au, Ag/AgCl, and Pt, respectively. Oligonucleotide miRNAs were purchased in the form of thiolated capture probes. The electrochemical biosensor was prepared according to Hakimian and Ghourchian [26]. Briefly, 25 mL of 9 mM AgNO_3_ and 5 mL of polyethyleneimine (PEI) were stirred during an increase in temperature to prepare PEI-Ag nanoparticles. 8 mL of 1 mM thiolated probe and 8 mL of phosphate-buffered saline (PBS) were mixed and poured onto the working electrode. Then, the working electrode was immersed in a solution of 1 M PBS buffer for 30 min, and then, 2 μL of the sample was mixed with 2 mL PBS and poured onto the electrode surface. 6 mL of positively charged PEI-Ag nanoparticles were poured onto the surface of the working electrode, and the adsorption process was performed on a negatively charged probe/sample compound. The electrodes were then placed in total RNA extracts of the leaves. To measure the electrochemical data of the biosensor, cyclic voltammetry curves were obtained by scanning in the range of 0.5 to − 0.5 V and a sweeping rate of 0.2 V/s.

**Table 1:** List of miRNAs investigated in this study

Related miRNA	miRNA sequence
miRNA156a	5′—UGACAGAAGAGAGUGAGCAC—3′
miRNA166i	5′—UCGGACCAGGCUUCAUUCUC—3′
miRNA399g	5′—AGGGCUUCUCUCCAUUGGCAGG—3′
miRNA477b	5′—CUCUCCCUCAAAGGCUUCUG—3′

### Machine learning method

Statistical regression models generally cannot find complex relationships between input and output variables in high-dimension problems. However, machine learning techniques can learn the relationships in datasets with hundreds of model inputs and outputs. A dataset was constructed to train machine learning methods with the experimental data gathered at different stress levels. Since 48 pots were used to investigate the effects of stresses and the data were gathered five times, the number of samples in the dataset was 240. Moreover, the total number of features was 13, including three morphological, six physiological/biochemical, and four miRNA features. SVM was used in this study to predict the type and severity of plant stress by having the values of experimental data as inputs. Two essential variables significantly influence the SVM performance: kernel type and kernel parameter (*γ*) [49]. Linear, polynomial, Gaussian, and sigmoid kernels are conventional kernel types in SVM. The value of *γ* was optimized using sophisticated metaheuristic optimization methods. As evolutionary-based and swarm intelligence-based methods, respectively, GA and PSO were utilized to optimize the kernel parameter of the SVM machine learning method to obtain a reliable machine for predicting plant stress having its characteristics.

GA involves a set of random chromosomes that describe possible solutions for the optimization problem. During generations, chromosomes with better values of fitness function can survive and crossover to create new offspring in the subsequent generations that are likely closer to the solution. The fitness function in this study is minimizing the error of predicting the plant stress using SVM. The maximum number of iterations, population size, and crossover percentage of GA were considered 500, 100, and 0.5, respectively. As another optimization technique, PSO includes a set of randomly-defined particles that move toward the solution in each iteration. The moving force of the particles in the search space is provided by a velocity vector. The particles close to the best positions (points with higher values of fitness function) will have a slow velocity, while the others will reach the best positions with higher velocities. After a number of iterations, all the particles approach the optimal point. The maximum number of iterations, population size, initial inertia weight, and cognitive acceleration in PSO were considered 100, 200, 1, and 1, respectively. The procedure of optimizing the SVM model using GA and PSO was similar to the one we used in our previous work on using optimization methods to tune the kernel parameter of SVM to obtain a reliable decision-making unit for a durable electrochemical nitrate biosensor [5].

Cooperative game theory is a reliable feature selection method in multivariate problems which was utilized in this work to determine the importance of morphological and physiological/biochemical features, as well as miRNA concentration, in the prediction of the plant stress response. There are variables with various importances in predicting the model outputs in datasets obtained from nature-based systems [69]. The cooperative game theory evaluates the amount of shared information (i.e., power or importance) by the model inputs using the Banzhaf power index [4, 70]).

A code written in the MATLAB R2018b programming environment was provided in this study to implement the machine learning and optimization algorithms. Five-fold cross-validation was used for investigating the performance of the algorithms based on the mean squared error (MSE) (Eq. 4) and coefficient of determination (*R*^2^) (Eq. 5)

4 $${\text{MSE}} = \frac{1}{n}\sum\limits_{i = 1}^{n} {(x_{{\text{p}}} - x_{{\text{o}}} )^{2} }$$

5 $$R^{2} = 1 - \frac{{\sum\limits_{i = 1}^{n} {(x_{{\text{p}}} - x_{{\text{o}}} )^{2} } }}{{\sum\limits_{i = 1}^{n} {(x_{{\text{o}}} - \overline{x}_{{\text{o}}} )^{2} } }}$$

where *x*_o_ is the severity of the applied stress to the plant, *x*_p_ is the predicted value of the severity using machine learning, and *n* is the number of samples.

## Results and discussion

Previous studies have revealed the remarkable influence of salinity and drought stress on the morphological and physiological characteristics of cucumber plants. However, specific detection of the stress can be challenging since plant response to stress can be complex and results in delayed response to the source of the stress. According to the objectives of this study, machine learning is used along with some miRNAs up- or down-regulated in cucumber plants for the specific detection of the source of stress shortly after applying the salinity and drought treatments. The characteristics are divided into three groups to investigate their individual effects on the prediction of the source and severity of the stress in cucumber plants: morphological, physiological/biochemical, and miRNA concentration characteristics. A dataset was created to implement the machine learning algorithms. The raw data collected during the experiment is brought in Additional file 1: Table S1 in the Additional file information.

### Performance of morphological characteristics in predicting plant stresses

Table 2 shows the performance of morphological variables in the prediction of plant stresses investigated in this study. As can be seen in the table, the morphological variables could not predict the type and severity of the plant abiotic stresses because of the low *R*^2^ and high MSE values. The highest prediction performance of the SVM model when both stresses were applied to the plants was 0.59, which was achieved for an SVM model with a Gaussian kernel. The morphological variables used in this study were image textural features examined by probability-density functions on GLCM. These features were extracted from the leaf images captured by an image acquisition system and transferred to a computer for further analysis. Although GA and PSO, as the metaheuristic optimization methods, could influence the performance of the machine in predicting the plant stresses, this influence was not sufficient to consider variables extracted from image processing useful for the prediction of plant abiotic stresses in cucumber plants. The highest prediction performance of the SVM model optimized with metaheuristic methods when both stresses were applied to the plants was 0.61, which was achieved for the SVM-GA model with a Gaussian kernel.

**Table 2:** Performance evaluation of morphological variables in the prediction of plant stress

Kernel type	Stress type	Model					
		SVM		SVM-GA		SVM-PSO	
		MSE	*R*^2^	MSE	*R*^2^	MSE	*R*^2^
Linear	Drought	1.52	0.56	1.43	0.59	1.46	0.58
	Salinity	1.46	0.58	1.43	0.59	1.28	0.64
	Drought + Salinity	1.94	0.42	1.88	0.44	1.88	0.44
Polynomial	Drought	1.46	0.58	1.46	0.58	1.43	0.59
	Salinity	1.64	0.52	1.61	0.53	1.58	0.54
	Drought + Salinity	1.97	0.41	1.88	0.44	1.97	0.41
Gaussian	Drought	1.85	0.45	1.79	0.47	1.79	0.47
	Salinity	1.67	0.51	1.58	0.54	1.67	0.51
	Drought + Salinity	1.43	0.59	1.37	0.61	1.43	0.59
Sigmoid	Drought	1.43	0.59	1.43	0.59	1.43	0.59
	Salinity	1.76	0.48	1.64	0.52	1.76	0.48
	Drought + Salinity	1.94	0.42	1.85	0.45	1.88	0.44

**Fig. 2:**
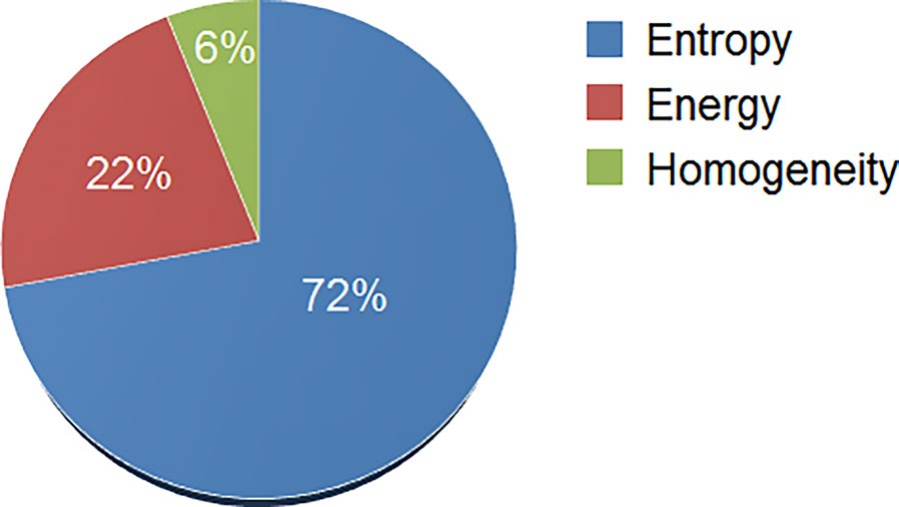
The amount of information shared by morphological variables in predicting the plant stress

Figure 2 shows that based on the results of cooperative game theory, entropy and homogeneity shared the highest and lowest amount of information among morphological variables in the prediction of plant stress, respectively. The leaves of the control plants that grew under optimal conditions were healthy and colorful, with high levels of entropy. Treated plants with salinity and drought had lower complexity in surface structure, and therefore, the entropy of their images decreased. Over time, the leaves of the control plants became darker green in color, and the energy of their images decreased [68]. In contrast, the yellowish appearance in the leaves of the treatment plants resulted in an increase in the energy levels in their images. Moreover, the local homogeneity of the images belonging to control plants decreased as they became colorful during growth, with different shades of green. Nonetheless, the treated plants, due to their uniform color, exerted higher homogeneity values in their images. Although the treated plants showed different morphological variables than the control plants, these differences were unreliable for specific detection of the type and severity of stresses using image processing (Table 2). Other morphological traits, e.g., plant height, shoot weight, and root weight, although generally influenced by increasing the severity of stress, this influence is not specific to the type of stress, as discussed in previous investigations [2, 35, 66].

### Performance of physiological/biochemical characteristics in predicting plant stresses

Table 3 shows the performance of combined physiological and biochemical variables in the prediction of plant stresses investigated in this study. According to the table, a machine with inputs containing physiological and biochemical variables could perform better than one with morphological variables in specific detection of stress. In this situation, the highest prediction performance of the single SVM model when both stresses were applied to the plants was 0.79, which was achieved for a model with a linear kernel. Metaheuristic optimization methods increased this performance up to 0.82, obtained for the SVM-PSO model. Generally, SVM-PSO provided better results than SVM-GA during the modeling process.

**Table 3:** Performance evaluation of physiological/biochemical variables in the prediction of plant stress

Kernel type	Stress type	Model					
		SVM		SVM-GA		SVM-PSO	
		MSE	*R*^2^	MSE	*R*^2^	MSE	*R*^2^
Linear	Drought	0.98	0.73	0.91	0.76	0.90	0.76
	Salinity	1.20	0.66	1.13	0.68	1.18	0.67
	Drought + Salinity	0.82	0.79	0.80	0.79	0.75	0.82
Polynomial	Drought	1.37	0.60	1.31	0.62	1.30	0.63
	Salinity	1.13	0.68	1.07	0.70	1.04	0.71
	Drought + Salinity	1.17	0.67	1.15	0.68	1.07	0.70
Gaussian	Drought	0.94	0.75	0.92	0.75	0.88	0.77
	Salinity	0.92	0.75	0.87	0.77	0.90	0.76
	Drought + Salinity	1.28	0.63	1.19	0.66	1.27	0.64
Sigmoid	Drought	1.10	0.69	1.07	0.70	1.08	0.70
	Salinity	1.13	0.68	1.07	0.70	1.04	0.71
	Drought + Salinity	1.01	0.72	0.98	0.73	0.98	0.73

**Fig. 3:**
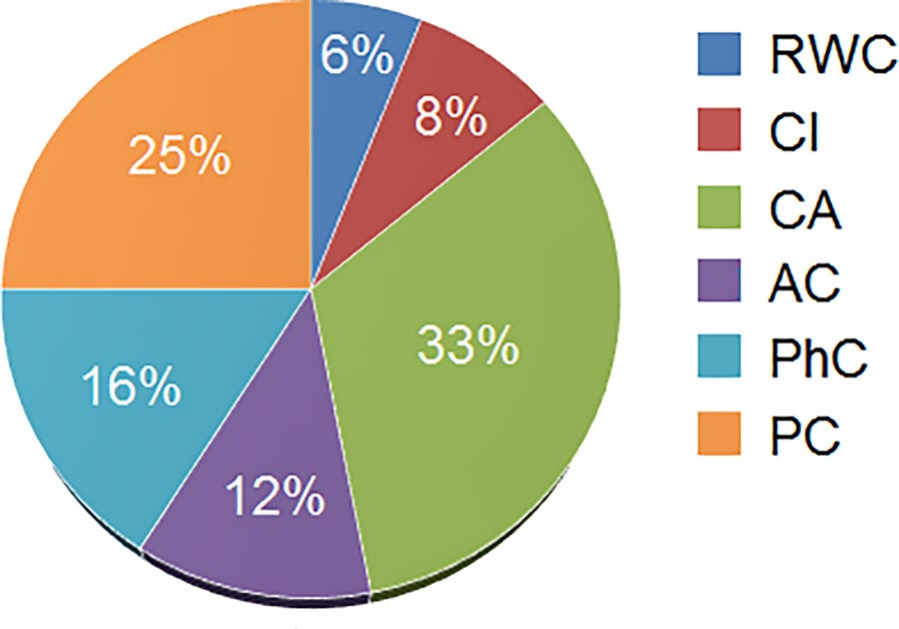
The amount of information shared by physiological/biochemical variables in predicting the plant stress

The results of cooperative game theory revealed that CA (33%) and RWC (6%) shared the highest and lowest amount of information among physiological and biochemical variables in predicting plant abiotic stresses, respectively (Fig. 3). Other variables had scores between these two values. RWC is an essential indicator for investigating plant water status and dehydration tolerance [41]. A remarkable decrease in the RWC of cucumber plant leaves might be due to the unavailability of water in the plant soil/root system [36, 59]. However, the results of this study show RWC had the lowest score in the prediction of stress. The possible reason is that various sources of stress have similar effects on RWC, and the prediction of the type and severity of the stress is challenging using only RWC as the model input.

During the experiments, the CA values in treatment plants were remarkably greater than those of control plants. [30] have reported similar results on the increased enzyme activity due to abiotic stresses. The complex enzymatic antioxidant system in plants affects oxygen species production during their exposure to abiotic stresses [27]. Similar trends were observed for AC, PhC, and PC, so these values were higher in the plants faced with stress compared to the control. Previous studies have shown positive correlations between abiotic tolerance and changes in antioxidant activity [15]. In fact, when plants are under stress conditions, antioxidative enzyme activities protect them against stress as a defense mechanism [63]. Studies have shown that younger plants express better protection against stress by increasing the antioxidant enzymatic activity [54].

CI, which is an essential plant physiological characteristic, decreased at severe stress levels. CI is positively correlated with photosynthetic activity [45, 72], and its decrement under abiotic stresses is considered an indicator of oxidative stress [53]. CI exerted an increasing trend in the control plant leaves as they became darker green in color over time, while CI in the treatment plants decreased, which was indicated by the yellowish appearance due to water deficiency.

### Performance of miRNA concentration characteristics in predicting plant stresses

As can be seen in Tables 2, 3, the prediction performance of machines trained by plant morphological and physiological/biochemical characteristics is not suitable for actual field situations since a reliable machine with high *R*^2^ and low MSE values is required by the farmers and agricultural specialists to determine the type and severity of stress correctly. Several studies have shown that the expression of miRNA156a, miRNA166i, miRNA399g, and miRNA477b changes in a tissue-specific behavior when cucumber plants are under abiotic stresses [18]. To encounter the problem during the prediction of stress, we tried to develop an intelligent model in which machine learning methods link the leaf miRNA concentration (or, in other words, expression) measured by an electrochemical biosensor to the stress. In this situation, portable electrochemical biosensors introduced in the literature that are capable of measuring the concentration of miRNAs at fM levels can be helpful for the accurate prediction of plant stresses. Moreover, features selection methods that determine the importance of each input in the prediction of model outputs can determine the contribution, i.e., effectiveness, of miRNAs to the plant stresses. This means that these methods provide us with valuable information about the miRNAs that should be measured by the biosensor to detect plant stress at early stages.

Table 4 shows the performance of miRNA concentrations in the prediction of plant stresses investigated in this study. Based on the table, miRNA concentrations were able to predict the type and severity of the stress with acceptable performance. The table also shows the type of optimization method to optimize the parameters of the SVM model has significant effects on the model performance. The most promising results (*R*^2^ = 0.99 and MSE = 0.23) were obtained by SVM-GA for the prediction of the type and severity of stress when the plants are faced with both salinity and drought conditions simultaneously.

**Table 4:** Performance evaluation of miRNA concentrations in the prediction of plant stress

Kernel type	Stress type	Model					
		SVM		SVM-GA		SVM-PSO	
		MSE	*R*^2^	MSE	*R*^2^	MSE	*R*^2^
Linear	Drought	0.31	0.96	0.21	0.99	0.25	0.98
	Salinity	0.65	0.84	0.62	0.85	0.60	0.86
	Drought + Salinity	0.31	0.96	0.23	0.99	0.24	0.98
Polynomial	Drought	0.59	0.86	0.51	0.89	0.55	0.88
	Salinity	0.68	0.83	0.64	0.85	0.66	0.84
	Drought + Salinity	0.64	0.85	0.59	0.86	0.56	0.87
Gaussian	Drought	0.43	0.92	0.42	0.92	0.39	0.93
	Salinity	0.51	0.89	0.51	0.89	0.46	0.91
	Drought + Salinity	0.58	0.87	0.53	0.88	0.57	0.87
Sigmoid	Drought	0.30	0.96	0.22	0.99	0.24	0.98
	Salinity	0.44	0.91	0.35	0.94	0.42	0.92
	Drought + Salinity	0.47	0.90	0.45	0.91	0.40	0.93

As expected, the miRNA concentrations altered toward the stresses. If not, the machine was unable to predict the stress with reliable performance. Some of the miRNAs were induced toward the stress conditions, while others were inhibited. From the perspective of machine learning approaches, it does not matter which miRNA is up-regulated and which one is down-regulated. What matters is that their response to the stress is specific, and the machine can discover this specificity. Similarly, various miRNAs are reported to act specifically toward metabolic activities in plants and animals [1, 80, 83]. Moreover, although a search in the literature shows that investigations on miRNA concentration using biosensors mainly belong to studies on humans [38, 55], studies on using biosensors for plant miRNA concentration have been growing rapidly since 2015s [3, 14, 40, 56].

**Fig. 4:**
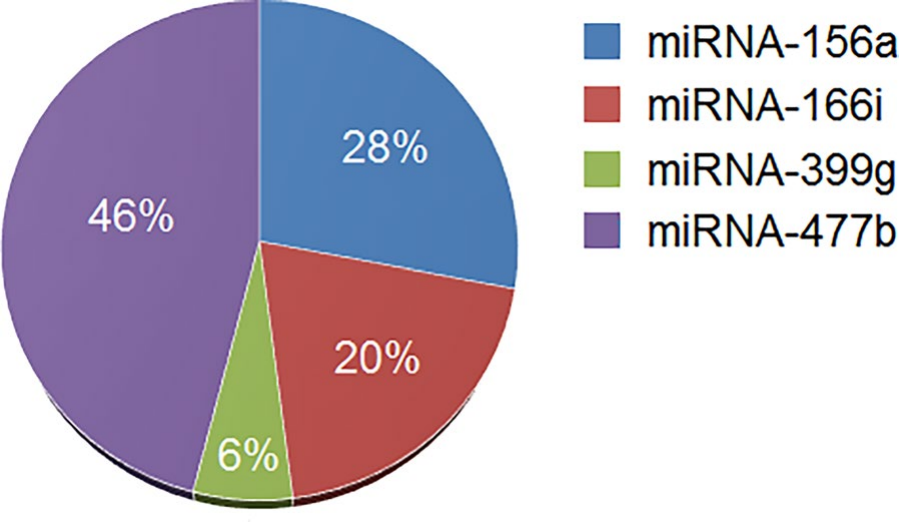
The amount of information shared by miRNA concentrations in predicting the plant stress

The main hypothesis that its correctness was proved in this study was that the concentration of some miRNAs extracted from cucumber plant leaves using a biosensor combined with the results of machine learning methods might be a specific marker of plant stress. However, the role of all miRNAs is not similar in the prediction task. As shown in Fig. 4, miRNA-477b concentration exerted the greatest contribution to the correct prediction of salt and drought stresses using machine learning. Therefore, among the miRNAs that are involved in plant abiotic stress, miRNA-477b concentration had the highest correlation with stress severity in cucumber plants. After that, miRNA-156a and miRNA-166i had rather similar importance in the prediction of stress, and miRNA-399 g had the lowest contribution.

miRNA-477 is associated with zeatin-O-glycosyltransferase regulation in leaf tissue. Expression of this miRNA is antagonistic to the respective gene under stress conditions. Previous studies have also reported that under mild and severe stress conditions, miRNA-477 and its target gene are both up-regulated and down-regulated, respectively. Zeation-O-glucosyltransferase is involved in cytokinin homeostasis and maintains the auxin/cytokinin ratio for plant growth regulation [61].

miRNA-156 is one of the well-studied plant miRNAs. It has essential functions in plant development, especially in leaf and flower development [84]. It targets Squamosa promoter-binding protein-like (SPL) transcription factors and, therefore, affects plant developmental timing [76]. Overexpression of this miRNA results in the fast establishment of rosette leaves and a rather delay in the flowering stage [65]. Besides, nitrogen deficiency in the environment induces the expression of miRNA-156 in plants [39].

An essential miRNA that controls leaf development in a wide variety of plants is miRNA-166 [46]. miRNA-166 mediates leaf morphogenesis by targeting the gene family that involves the class-III homeodomain leucine zipper transcription factor [34]. Overexpression of miRNA-166-resistant PHABULOSA mutants causes unusual changes in the leaf developmental stage. Furthermore, the overexpression of plant miRNA-166 results in vascular cell differentiation, followed by the production of a more vascular system with expanded xylem tissue [37].

miRNA-399 is an essential miRNA in plants that is highly induced in plant tissues under phosphate stress [9]. miRNA399 expression in transgenic seedlings results in suppressing the putative ubiquitin-conjugating enzyme transcript under high inorganic phosphate conditions [78]. Since miRNA-399 g had a negligible contribution to stress prediction compared to the other three miRNAs using machine learning algorithms, it was omitted from the dataset, and SVM-GA was used to predict the type and severity of the study stresses by having only the concentration of miRNA-156a, miRNA-166i, and miRNA-477b. The results showed that the *R*^2^ and MSE of prediction when both stresses were applied to the cucumber plants were 0.98 and 0.25, respectively. This indicates that developing a biosensor that measures the concentration of these three miRNAs is reliable for in-field stress determination.

## Conclusions

An effort to introduce a novel method to detect the type and severity of two main abiotic stresses, i.e., salinity and drought, in cucumber plants is reported in this paper. Treatments were selected in levels to apply a range of mild to rather severe stress conditions. The results proved that in all stress conditions, the miRNA biosensor equipped with a machine learning algorithm optimized by metaheuristic methods, that can measure the concentration of miRNAs shown effective in the plant stress response based on previous studies, is suitable to detect stress in cucumber plants (*R*^2^ = 0.99). This reveals that compared to plant morphological and physiological features, miRNA concentrations are more reliable features for providing us with valuable information about plant abiotic diseases at early stages. Therefore, extracting the plant morphological and physiological features, which are usually time-consuming and require transferring the samples to well-equipped laboratories, to provide a reliable understanding of plant stress status will no longer be needed if a miRNA biosensor is available to measure the miRNA concentrations.

As a sensor based on a three-electrode platform, the biosensing method to measure the concentration of miRNAs is portable and can be easily used by farmers and agricultural specialists. The biosensor can be connected to a laptop in which the machine learning algorithm is running. Even internet-of-things (IoT) solutions to wirelessly transfer the electrochemical data of the biosensor to a host computer can be implemented to increase the functionality of the method described in this study for real in-field applications.

### Supplementary Information

**Additional file 1: Table S1.** Cucumber plant characteristics in various drought and salinity stress levels. W_0_ to W_3_ and S_0_ to S_3_ indicate drought and salinity stress levels, respectively, which are described in the main manuscript.

## Data Availability

The datasets used and analyzed during the current study are available from the corresponding author upon reasonable request.
