# Supplementary material for: Machine learning provides specific detection of salt and drought stresses in cucumber based on miRNA characteristics
Source: Plant Methods. 2023 Nov 8;19:123. doi: 10.1186/s13007-023-01095-x (PMC10631058; doi:10.1186/s13007-023-01095-x)
Supplement: Supplementary file 1 — Additional file 1: Table S1. Cucumber plant characteristics in various drought and salinity stress levels. W0 to W3 and S0 to S3 indicate drought and salinity stress levels, respectively, which are described in the main manuscript. [file 13007_2023_1095_MOESM1_ESM.docx]

# Additional file information

# Machine learning provides specific detection of salt and drought stresses in cucumber based on miRNA characteristics

Parvin Mohammadi^1^, Keyvan Asefpour Vakilian^2*^

^1^ Department of Agrotechnology, College of Abouraihan, University of Tehran, Tehran, Iran

^2^ Department of Biosystems Engineering, Gorgan University of Agricultural Sciences and Natural Resources, Gorgan, Iran

* Email address of KA for correspondence: [keyvan.asefpour@gau.ac.ir](mailto:keyvan.asefpour@gau.ac.ir)

Table S1. Cucumber plant characteristics in various drought and salinity stress levels. *W*_0_ to *W*_3_ and *S*_0_ to *S*_3_ indicate drought and salinity stress levels, respectively, which are described in the main manuscript.

| miRNA-477b  (fM) | miRNA-399g  (fM) | miRNA-166i  (fM) | miRNA-156a  (fM) | PC  (mg g^-1^ Fwt) | PhC  (mg GAE g^-1^ Fwt) | AC  (A530 g^-1^ Fwt) | CA  (units mg^-1^) | CI  (%/%) | RWC  (%) | Homogeneity | Energy | Entropy | Salinity  stress | Drought stress |
| --- | --- | --- | --- | --- | --- | --- | --- | --- | --- | --- | --- | --- | --- | --- |
| 1334±238 | 83±22 | 361±62 | 17±5 | 8.32±2.14 | 0.31±0.09 | 0.72±0.13 | 0.31±0.09 | 461.3±95.1 | 94.1±3.7 | 0.376±0.141 | 0.205±0.065 | 0.657±0.095 | *S*_0_ | *W*_0_ |
| 1255±288 | 94±25 | 355±73 | 82±10 | 8.76±1.38 | 0.38±0.08 | 0.81±0.21 | 0.32±0.09 | 394.2±54.0 | 93.2±3.1 | 0.395±0.109 | 0.264±0.048 | 0.590±0.134 | *S*_1_ |  |
| 733±192 | 94±36 | 389±94 | 368±39 | 9.21±1.13 | 0.38±0.08 | 1.12±0.27 | 0.35±0.08 | 331.1±84.4 | 94.3±1.8 | 0.391±0.086 | 0.257±0.059 | 0.575±0.106 | *S*_2_ |  |
| 625±106 | 120±37 | 358±100 | 573±145 | 9.35±2.45 | 0.42±0.09 | 1.19±0.22 | 0.36±0.09 | 322.2±74.6 | 92.8±3.3 | 0.451±0.132 | 0.288±0.077 | 0.509±0.085 | *S*_3_ |  |
| 1162±175 | 122±21 | 355±48 | 23±6 | 8.27±2.72 | 0.38±0.09 | 0.70±0.23 | 0.33±0.11 | 391.0±52.2 | 91.8±1.2 | 0.403±0.095 | 0.253±0.095 | 0.625±0.164 | *S*_0_ | *W*_1_ |
| 1008±276 | 102±30 | 393±83 | 111±33 | 12.74±2.37 | 0.39±0.08 | 0.81±0.17 | 0.31±0.08 | 364.8±80.8 | 92.0±2.8 | 0.437±0.114 | 0.286±0.031 | 0.608±0.054 | *S*_1_ |  |
| 833±269 | 122±44 | 449±79 | 328±76 | 13.49±3.23 | 0.38±0.11 | 1.21±0.22 | 0.37±0.08 | 345.2±63.2 | 88.9±2.7 | 0.482±0.146 | 0.295±0.062 | 0.532±0.208 | *S*_2_ |  |
| 893±148 | 127±39 | 452±88 | 643±155 | 10.21±3.07 | 0.44±0.09 | 1.39±0.42 | 0.36±0.12 | 271.0±94.5 | 86.2±3.6 | 0.508±0.128 | 0.322±0.078 | 0.454±0.131 | *S*_3_ |  |
| 1227±155 | 134±19 | 419±81 | 21±4 | 7.83±2.80 | 0.42±0.08 | 0.83±0.18 | 0.37±0.09 | 352.5±66.8 | 84.1±2.5 | 0.389±0.108 | 0.246±0.064 | 0.602±0.104 | *S*_0_ | *W*_2_ |
| 1100±206 | 118±26 | 487±108 | 90±30 | 8.36±3.18 | 0.46±0.08 | 0.82±0.25 | 0.40±0.12 | 321.1±90.2 | 85.2±3.1 | 0.441±0.076 | 0.273±0.050 | 0.544±0.077 | *S*_1_ |  |
| 927±172 | 104±41 | 418±85 | 374±82 | 12.23±2.72 | 0.50±0.09 | 1.19±0.28 | 0.43±0.14 | 287.3±83.3 | 83.9±2.7 | 0.438±0.113 | 0.234±0.037 | 0.487±0.182 | *S*_2_ |  |
| 649±100 | 142±48 | 462±173 | 532±193 | 12.85±3.77 | 0.39±0.10 | 1.66±0.33 | 0.49±0.09 | 238.2±74.7 | 88.8±4.0 | 0.497±0.160 | 0.295±0.053 | 0.398±0.132 | *S*_3_ |  |
| 956±143 | 141±32 | 500±188 | 32±4 | 10.99±4.22 | 0.45±0.08 | 0.92±0.20 | 0.39±0.11 | 308.9±62.1 | 87.2±2.2 | 0.375±0.087 | 0.273±0.075 | 0.588±0.065 | *S*_0_ | *W*_3_ |
| 857±98 | 142±49 | 477±142 | 117±28 | 12.19±3.40 | 0.46±0.08 | 0.99±0.32 | 0.40±0.09 | 274.0±86.9 | 89.4±2.0 | 0.412±0.133 | 0.290±0.067 | 0.477±0.107 | *S*_1_ |  |
| 761±134 | 150±32 | 431±108 | 299±66 | 13.32±4.01 | 0.40±0.09 | 1.88±0.37 | 0.42±0.12 | 232.9±97.4 | 86.1±1.9 | 0.429±0.193 | 0.311±0.053 | 0.325±0.121 | *S*_2_ |  |
| 585±122 | 173±37 | 505±162 | 792±243 | 14.18±4.37 | 0.59±0.12 | 1.70±0.32 | 0.51±0.10 | 219.3±47.6 | 81.8±3.2 | 0.556±0.135 | 0.307±0.104 | 0.294±0.144 | *S*_3_ |  |

RWC: relative water content, CI: chlorophyll index, CA: catalase activity, AC: anthocyanin content, PhC: phenolic compounds, PC: proline content

Data are the means ± SD, averaged from three replications for five measurements with three-day intervals.
